# Microglial responses around intrinsic CNS neurons are correlated with axonal regeneration

**DOI:** 10.1186/1471-2202-11-13

**Published:** 2010-02-05

**Authors:** Bahman N Shokouhi, Bernadette ZY Wong, Samir Siddiqui, A Robert Lieberman, Gregor Campbell, Koujiro Tohyama, Patrick N Anderson

**Affiliations:** 1 Department of Cell and Developmental Biology, University College London, Gower Street, London, WC1E 6BT, UK; 2 Centre for Electron Microscopy and Bioimaging Research, Laboratory for Nano-Neuroanatomy, Iwate Medical University School of Medicine, 19-1 Uchimaru, Morioka, Iwate, 020-8505 Japan

## Abstract

**Background:**

Microglia/macrophages and lymphocytes (T-cells) accumulate around motor and primary sensory neurons that are regenerating axons but there is little or no microglial activation or T-cell accumulation around axotomised intrinsic CNS neurons, which do not normally regenerate axons. We aimed to establish whether there was an inflammatory response around the perikarya of CNS neurons that were induced to regenerate axons through a peripheral nerve graft.

**Results:**

When neurons of the thalamic reticular nucleus (TRN) and red nucleus were induced to regenerate axons along peripheral nerve grafts, a marked microglial response was found around their cell bodies, including the partial enwrapping of some regenerating neurons. T-cells were found amongst regenerating TRN neurons but not rubrospinal neurons. Axotomy alone or insertion of freeze-killed nerve grafts did not induce a similar perineuronal inflammation. Nerve grafts in the corticospinal tracts did not induce axonal regeneration or a microglial or T-cell response in the motor cortex.

**Conclusions:**

These results strengthen the evidence that perineuronal microglial accumulation (but not T-cell accumulation) is involved in axonal regeneration by intrinsic CNS and other neurons.

## Background

Axons in injured peripheral nerves regenerate vigorously whereas most intrinsic CNS neurons do not spontaneously regenerate their axons. However, some intrinsic CNS neurons, including those in the TRN and rubrospinal neurons, can be induced to regenerate their axons by the implantation of a segment of living peripheral nerve into the brain or spinal cord [1]. Axonal regeneration then occurs within the conducive environment of the nerve graft. Although successful axonal regeneration requires a suitable environment for the elongating axons, the vigour of axonal regeneration is determined by the cell body response to axotomy. The cell body response of intrinsic CNS neurons is generally less marked than that of motor or sensory neurons following peripheral nerve injury. Furthermore, only those populations of intrinsic CNS neurons that are capable of regenerating axons into nerve grafts mount a prolonged cell body response to axotomy [1].

There is increasing evidence that inflammatory responses in nerve trunks are important for axonal regeneration [2]. In addition, the cell body response to axotomy may be linked to the presence of inflammatory cells in the vicinity of the injured neurons. In response to nerve injury, microglia around motor neurons become activated, proliferate and migrate towards perikarya of the axotomized neurons [3-5] which they enwrap. Similarly, macrophage activation has been detected around dorsal root ganglion (DRG) neurons projecting into injured peripheral nerves [6-8]. A variety of genes related to inflammatory responses are upregulated in axotomised DRG and autonomic neurons [9,10]. In contrast, there is disagreement over whether there is usually microglial activation and/or an increase in microglial numbers around the cell bodies of axotomized intrinsic CNS neurons. No increase in numbers or activation of microglia was reported around cortical projection neurons following pyramidotomy [11], whereas following rubrospinal tract injury in the spinal cord, microglial activation in the red nucleus has been reported to be absent [12], minimal and transient [13], or noticeable [14,15]. Even in the latter case the inflammation was much more modest than that found around axotomised motor neurons. Thus the extent of perineuronal microglia activation correlates well with vigorous axonal regeneration. T-cells also accumulate in the vicinity of axotomized motor, sensory, and autonomic neurons [8,16,17]. However there are no data concerning the presence of T-cells around axotomized and/or regenerating intrinsic CNS neurons.

Inflammation around neuronal cell bodies enhances the regenerative responses of both DRG neurons [18,19] and retinal ganglion cells [20]. The accumulation of T-cells around perikarya in response to axotomy is generally believed to be neuroprotective [21-23]. The microglial and T-cell responses could also be involved in other aspects of the response to nerve injury, such as defence against infection [17,24].

If the microglial and T-cell responses around axotomized neurons are part of the mechanism by which axonal regeneration is stimulated, they should be found around intrinsic CNS neurons regenerating axons into a peripheral nerve graft, but probably not around intrinsic CNS neurons subject to axotomy alone. We have tested this hypothesis by implanting segments of peripheral nerve into the CNS of adult rats to induce axonal regeneration by intrinsic CNS neurons, and monitoring the responses of microglia and T-cells around regenerating versus non-regenerating neurons.

## Results

Antibody to β-thymosin and the OX42 antibody to the rat complement receptor 3/CD1l b antigen produced similar labelling of microglia, except that β-thymosin generally stained microglia more completely. The cell bodies of ramified microglia were generally better visualised with β-thymosin antibody, as were microglia in superficial layers of the cerebral cortex. However, stocks of β-thymosin antibody became depleted during the study, after which OX42 was used to detect microglia. The OX52 antibody to the rat CD6 antigen and the antibody to the T-Cell Receptor (TCR) produced identical surface staining of small rounded or oval cells about 4 μm diameter that were identified as T-cells. Negative controls showed faint background staining of neurons but not microglia or T-cells.

### Facial nerve injury causes microglial activation and T-cell accumulation in the facial nucleus

**Figure 1:**
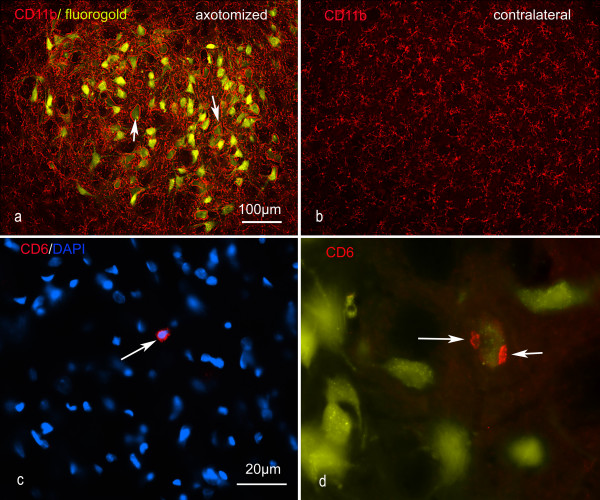
**Representative images of the inflammatory changes in the facial nucleus during axonal regeneration, one week following facial nerve transaction**. a, b: CD11b immunoreactivity for microglia is increased in the axotomized facial nucleus, and microglia enwrap the facial motor neurons, e.g. at arrows. The regenerating neurons were retrogradely labelled with fluorogold. c, d: CD6- positive T-cells accumulated in the injured motor nucleus (arrows). They had little cytoplasm but dense nuclei (c) and were sometimes clustered around neurons retrogradely labelled with fluorogold (d). The scale bar in (a) also applies to (b) and that in (c) also applies to (d).

It was first confirmed that microglial cells were activated and T-cells accumulated around vigorously regenerating neurons in adult rats. In 6 rats the facial nerve was transected just below the ear cartilage and fluorogold applied to the cut end in 2 of the animals. Microglial CD11b immunoreactivity was greatly enhanced in the axotomized nucleus 1 week after injury (Fig. 1a, b) and reactive microglial cells surrounded the retrogradely labelled and ATF3-positive facial motor neurons. ATF3 expression can be used to identify axotomized and regenerating neurons in the absence of retrograde label [25,26]. There were obviously increased numbers of CD6-positive and TCR -positive T-cells in the facial nucleus. In 3 control, unoperated rats the mean number of T-cells in sections of the facial nucleus was 1.3 +/- 1.1 per mm^2^. The number of CD6-positive T-cells in the axotomised facial nuclei of 6 rats 7 days after facial nerve transection was 20.35 +/- 3 per mm^2^, with a mean of 3.07 +/- 1.45 T-cells per mm^2 ^in the contralateral nucleus. The T-cells in the axotomized nucleus were sometimes clustered, or in close contact with motor neurons (Fig. 1c, d). Thus it was confirmed that a peripheral nerve injury that leads to vigorous axonal regeneration in adult rats provoked a perineuronal inflammatory response involving both microglia and T-cells.

### Thalamic lesions without a peripheral nerve graft induce a modest microglial reaction close to the injury site but no T-cell response in the TRN

**Figure 2:**
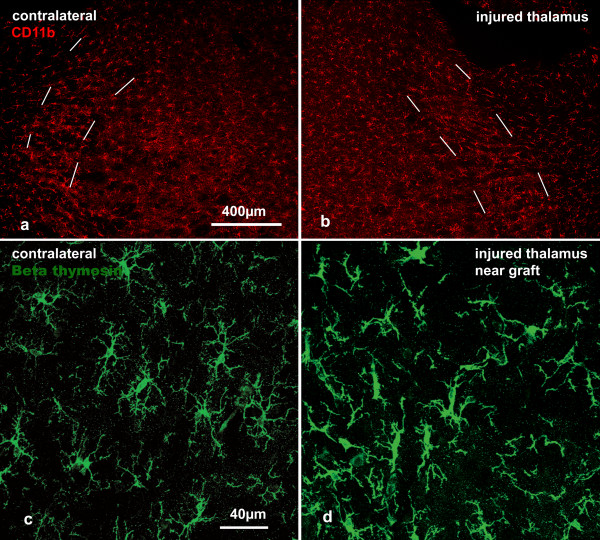
**Microglia in the TRN following stab wounds in thalamus**. a, b: Representative images of immunoreactivity for CD11b microglia in the TRN (between dashed lines) immediately rostral to the lesion 4 weeks after injury. There is little difference between the injured and contralateral sides. c, d: Confocal images showing signs of activation of β thymosin-+ microglia within the TRN within 500 μm of the lesion, 5 days following injury. The processes of microglia are thicker and less extensively branched than on the contralateral (uninjured) side. The scale bar in (a) also applies to (b) and that in (c) also applies to (d).

In animals with a stab injury to the dorsal thalamus, examined 5 days (n = 2), 2 weeks (n = 2) and 4 weeks (n = 3) post injury, the morphological signs of activation of microglia were mainly confined to the perilesion area and the TRN closest to the lesion (Fig. 2a-d); even those microglia within the TRN closest to the lesion remained ramified. The microglia in the TRN rostral to the lesion (containing cells projecting to the lesion site), and those in the contralateral TRN, showed no morphological signs of activation. Stab wounds to the thalamus had little effect on the number of CD6-positive T-cells in the TRN. A few T-cells were present in the lesion site and in the thalamus within 200 μm of the lesion at all times examined but T-cells were rarely identified in the TRN on the injured or contralateral sides.

### Cervical rubrospinal tract lesions induce a modest microglial reaction but no T-cell accumulation in the axotomized red nucleus

**Figure 3:**
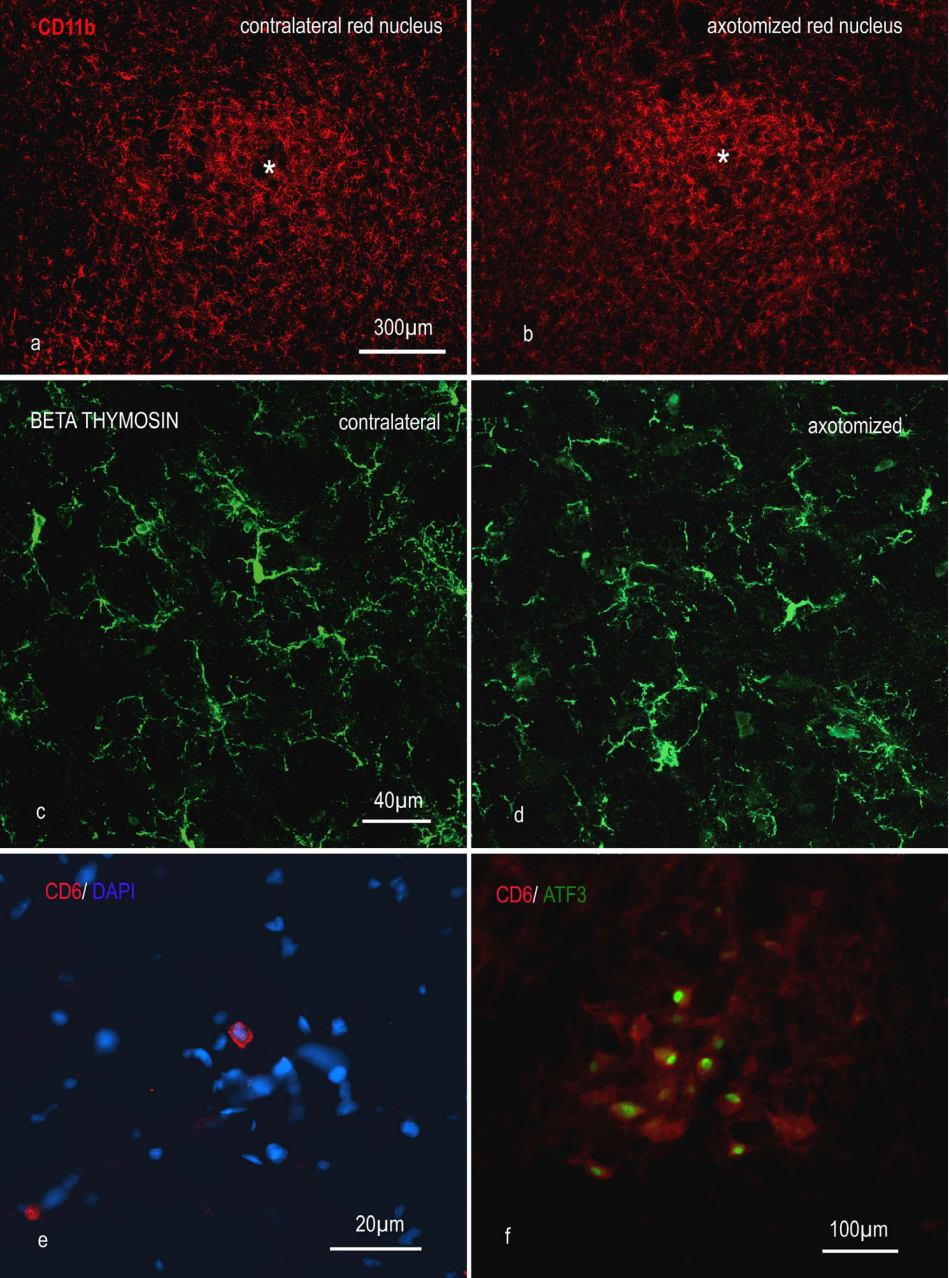
**Rubrospinal injury produces little microglial activation and no accumulation of T-cells in the red nucleus**. a, b: Representative images show there is little difference in CD11b immunoreactivity for microglia in the red nucleus (*) one week following axotomy. c, d: confocal images of β thymosin immunoreactive microglia in the red nucleus 3 weeks after injury. e: a rare CD6-positive T lymphocyte in the red nucleus of an unoperated rat. f: one week after injury no T lymphocytes can be identified in the red nucleus around the axotomized rubrospinal neurons, which can be recognised by their expression of ATF3 (green). Neuronal cytoplasm has been visualised by high gain in the red signal; this does not represent CD6 signal.

**Figure 4:**
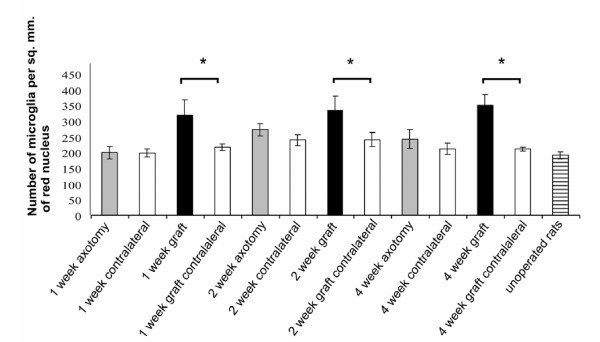
**Histogram showing the number of CD11b-positive microglia per mm^2 ^in the red nucleus following axotomy alone or insertion of a peripheral nerve graft into the rubrospinal tract**. Axotomy (grey columns) has little effect on microglial numbers whereas insertion of a peripheral nerve graft into the rubrospinal tract to induce axonal regeneration (black columns) produces a prolonged increase in the number of microglia within the red nucleus. The results are from 4 animals in each case (*P < 0.05).

The intact red nucleus contained microglia immunoreactive for CD11b and β-thymosin but very few T-cells. In 6 animals with section of the right cervical rubrospinal tract, but no nerve graft, killed at 1 week post operation, in 5 rats killed at 2 weeks post operation, and in 5 animals killed at 4 weeks post operation, there was little consistent qualitative difference in the microglial population of the axotomized or contralateral red nuclei (Fig. 3a-d). The number of microglia was counted in sections from the red nucleus of 4 animals at 1, 2 and 4 weeks after operation, stained for CD11b. In some sections at one week post operation there was a modest increase in immunoreactivity on the axotomized side but this was not reflected in the numbers of microglial cells present (Fig. 4). The number of CD11b-positive microglia in the red nucleus increased after 2 weeks but there was no significant difference between the axotomised and control sides. The microglia in the red nucleus of all animals had a resting, ramified morphology. No enwrapping of rubrospinal cell bodies was observed. Single T-cells were found in the red nucleus in two of the sections of the midbrain of animals of control rats (Fig. 3e) but none was found in an axotomised red nucleus (Fig. 3f).

### Corticospinal tract lesions without a peripheral nerve graft produce no microglial or T-cell response in the motor cortex

**Figure 5:**
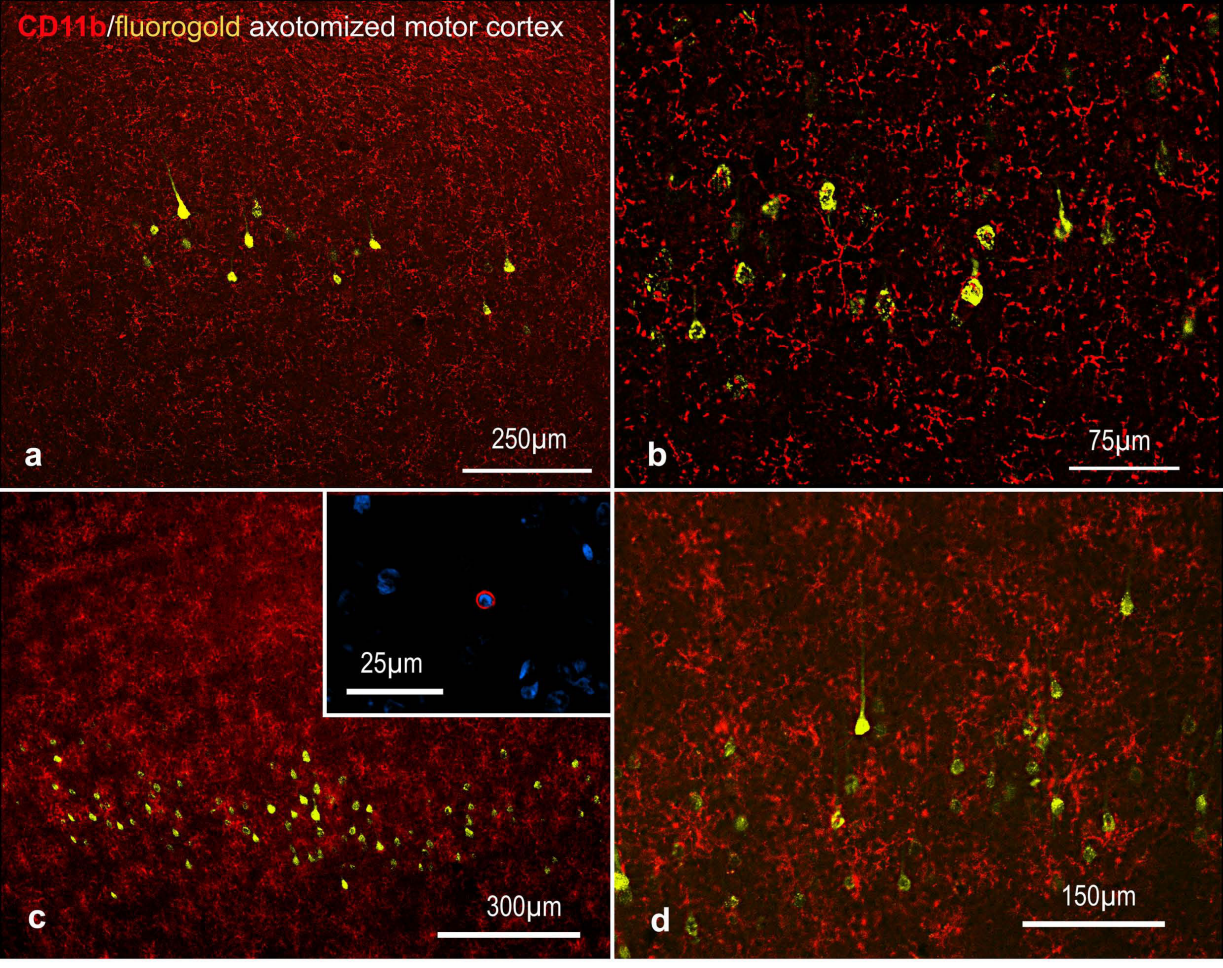
**CD11b immunoreactive (red) microglia in the motor cortex following cervical corticospinal tract injury**. Representative images of motor cortex - a, b: one week (deconvolved images); c, d: two weeks following corticospinal tract injury. The corticospinal neurons were retrogradely labelled with fluorogold applied to the spinal cord at the time of injury. There is little sign of microglial activation. The inset in (c) shows a CD6-positive T-cell in the motor cortex, but no accumulation of such cells was detected.

**Figure 6:**
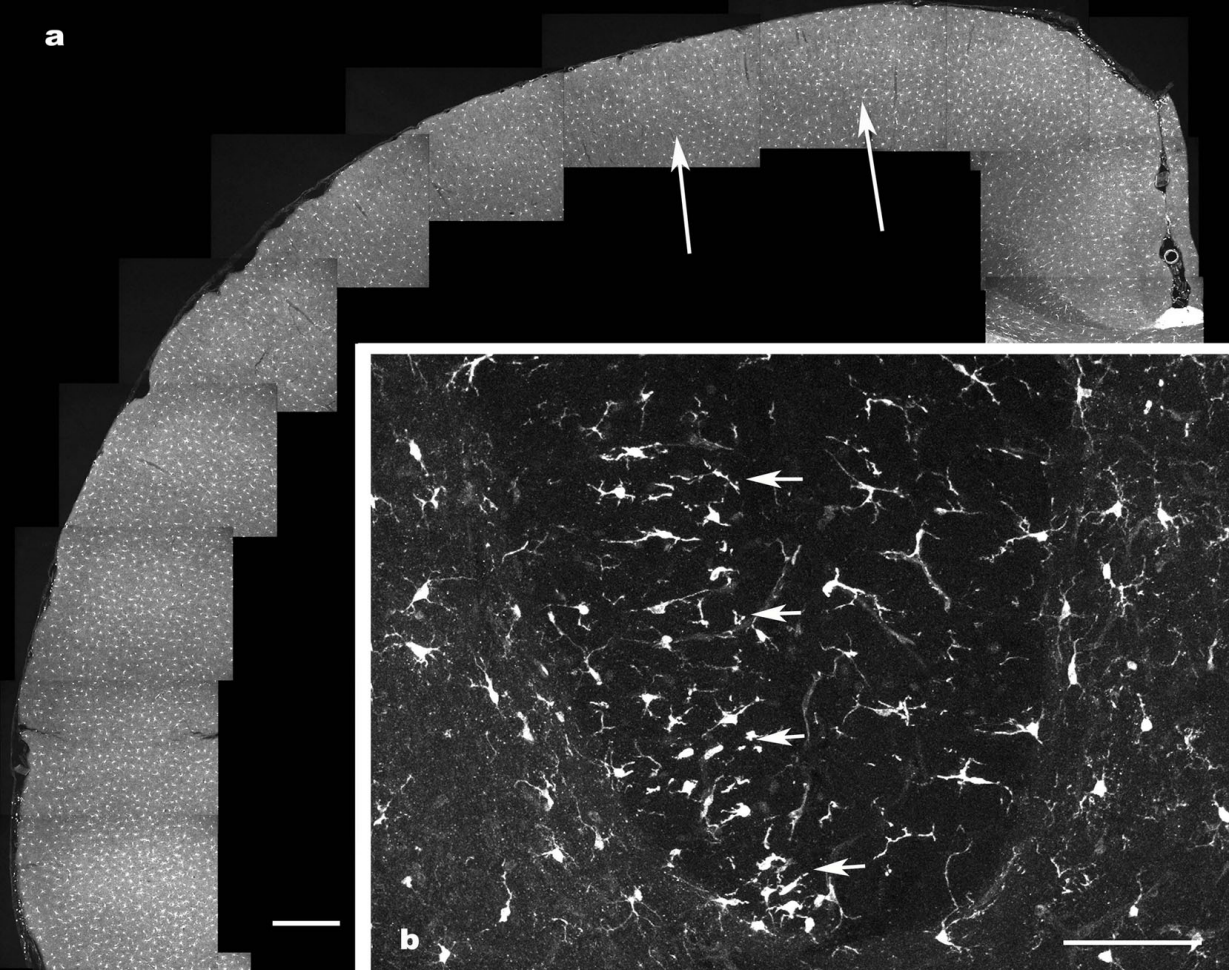
**β thymosin immunoreactivity in the motor cortex 7 days following unilateral pyramidectomy**. The panel (a) is a montage showing the absence of increased microglial staining in the motor cortex (arrowed) compared with adjacent cortex. The panel (b) shows the unilateral microglial reaction in the degenerating dorsal corticospinal tract (arrows) from the same animal. Both scale bars are 100 μm.

Bilateral dorsal corticospinal tract lesions, combined with retrograde tracing with fluorogold, were performed on 3 rats killed 1 week post operation and 3 rats killed at 2 weeks post operation and reacted for CD11b. No activation of CD11b-positive microglia was detected around the retrogradely labelled neurons in the upper or lower limb regions of the motor cortex (Fig. 5). Unilateral pyramidectomy was performed to achieve complete unilateral axotomy of corticospinal neurons in 5 rats, all killed 7 days after operation. No activation of microglia, identified using β-thymosin antibody, was detectable in the axotomised motor cortex (Fig. 6) and no differences could be detected in the density or morphology of the microglia in the motor cortex of the axotomized side compared with the contralateral side.

There was no significant difference in the number of T-cells in the motor cortex in response to bilateral corticospinal tract transection in the cervical spinal cord. In 3 control, unoperated rats, 0.8 +/- 1.0 CD6-positive T-cells were counted per field in layer 5. In 3 rats killed 2 weeks after bilateral corticospinal transection there were 0.6 +/- 0.4 T-cells per field in layer 5.

### Peripheral nerve graft experiments

Intrinsic CNS neurons regenerating axons into peripheral nerve grafts can only be unambiguously identified by retrograde labelling from the distal ends of the grafts. This can only be achieved reliably at 21 days or more after grafting, by which time regenerating axons have grown into and through the graft.

### Insertion of a living peripheral nerve graft into the thalamus induces regeneration of TRN neurons and inflammatory changes around the perikarya of the neurons with regenerating axons. Freeze-killed grafts had no such effects

Experiments in our laboratory and others have consistently shown that neurons in the TRN reliably regenerate axons into nerve grafts in the thalamus whereas few thalamic projection neurons do so [27-30]. This was confirmed in the present study.

In 13 rats killed between 5 days and 2 weeks after grafting there were, as expected, no retrogradely labelled neurons in the thalamus. However, in all these rats there was markedly increased immunoreactivity for CD11b or β thymosin in the TRN on the operated side, in regions rostral to the graft tip which project to the grafted area, compared to the contralateral TRN.

**Figure 7:**
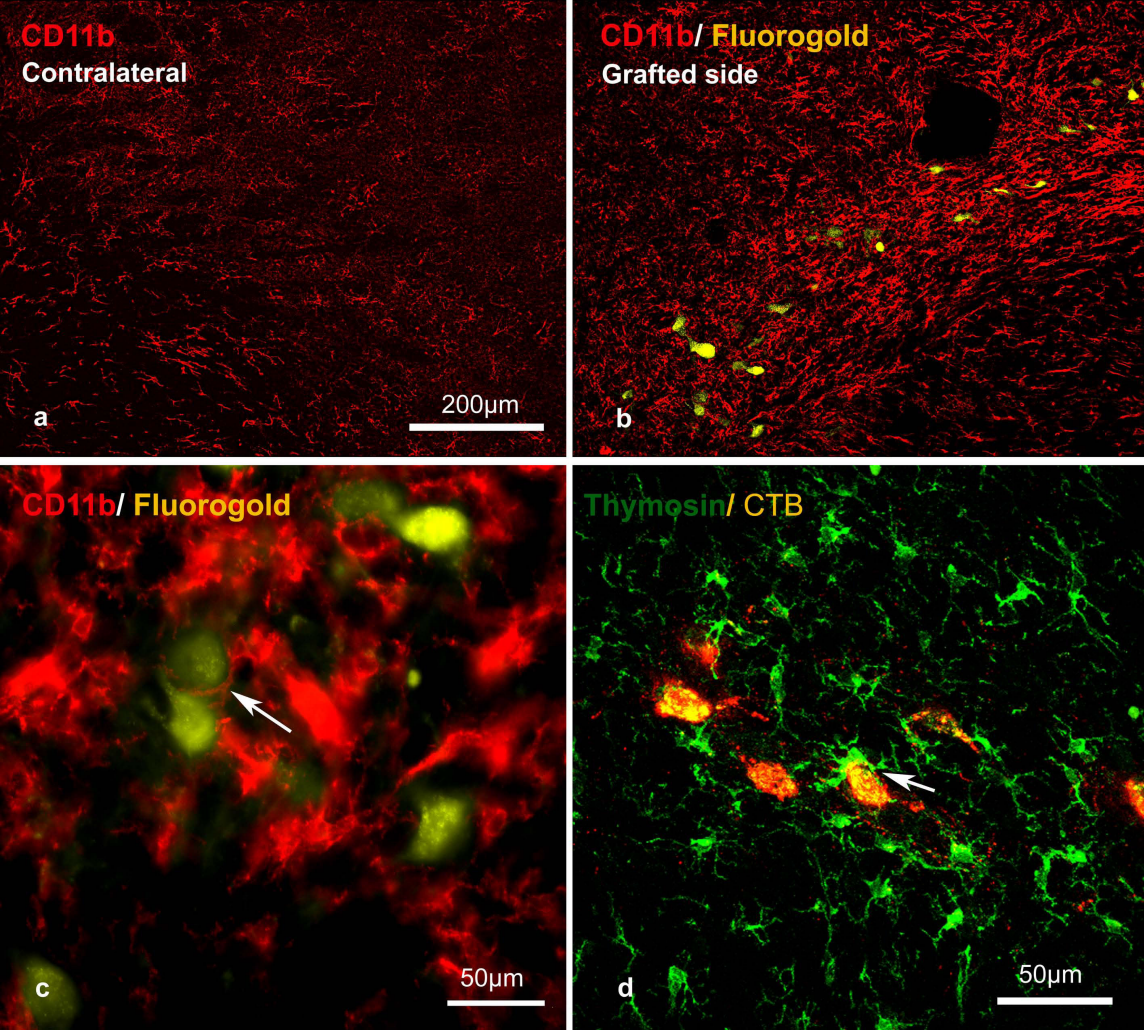
**Representative images of microglia in the TRN following nerve implantation into the thalamus**. (a and b) are deconvolved digital images, (c) is a non-deconvolved image and (d) is a confocal image. The neurons which regenerated axons into the grafts were retrogradely labelled with fluorogold (a, b and c) or CTB (d). There is clearly increased CD11b activity in the TRN around the retrogradely - labelled neurons which are regenerating axons (b) compared with the contralateral (uninjured) side (a). There was close contract between microglia and TRN neurons with regenerating axons (arrows in c and d). The scale bar in (a) also applies to (b).

In 5 rats with survival times of 3 weeks (n = 4) and 4 weeks (n = 1), retrograde labelling with CTB was successfully combined with β-thymosin immunohistochemistry (Fig. 7d). In 4 further rats at 4 weeks post operation, retrograde tracing with fluorogold was successfully combined with CD11b immunohistochemistry (Fig. 7a-c). In all of these rats retrogradely-labelled neurons were present in the TRN on the grafted side, at the level of and rostral to the graft tip. Immunoreactivity for β thymosin and CD11b was more intense in the TRN on the grafted side, including all regions where retrogradely labelled TRN neurons were identified (Fig. 7). The β thymosin and CD11b-positive activated microglia on the grafted side generally had shorter, thicker processes and larger cell bodies than their counterparts in the non-grafted side. Microglial processes wholly or partially enwrapped the cell bodies of some neurons with regenerating axons (i.e. retrogradely labelled cells; Fig 7c and 7d). Microglia were counted only in the sections of TRN of 3 rats with retrogradely labelled TRN neurons at 3-4 weeks post operation stained for β thymosin. 366 +/- 48 microglia per mm^2 ^were present on the grafted side compared with 247 +/- 59 per mm^2 ^on the contralateral side. Increased numbers of microglia were also seen near the graft/brain interface. Much smaller increases were found in the internal capsule. No significant differences were observed between the dorsal and ventral TRN.

**Figure 8:**
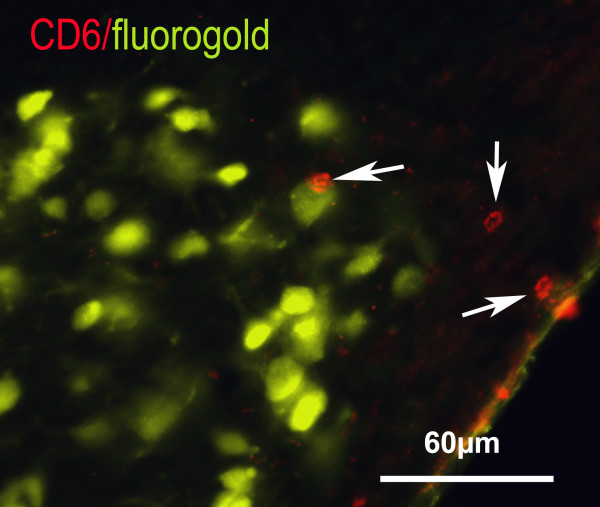
**CD6-positive cells (some arrowed) accumulate in the TRN close to a graft in the thalamus**. TRN neurons regenerating axons into the graft were retrogradely labelled with fluorogold. The space in the bottom right corner is a blood vessel.

Four weeks after implanting a tibial nerve graft into the thalamus, T-cells were abundant within the nerve graft, much more so than in stab wound lesion sites, and were present at the graft/brain interface. In sections that passed close to the grafts T-cells were also present in the TRN (a mean of 6.3 +/- 2.1 within the grafted TRN per section, n = 4) in regions where the cell bodies of fluorogold-labelled (regenerating) neurons were found (Fig. 8), and in adjacent areas of the dorsal thalamus. However only a few CD6-positive T-cells were found in the TRN rostral to the graft (where retrogradely labelled neurons were also found and the microglial reaction was strong) and they were rare in the TRN on the contralateral side (a mean of 0.66 +/- 0.58 T-cells within the TRN per section).

Freeze-killed grafts initially contain no living cells [31] and do not support the regeneration of axons by intrinsic CNS neurons [32,33]. In 3 rats given freeze-killed grafts into the thalamus and killed 4 weeks after operation, no retrogradely labelled neurons were found in the TRN and there was no increase in immunofluorescence for CD11b (data not shown).

Thus, provoking axonal regeneration of TRN neurons produced a perineuronal microglial response around all the regenerating cells and an accumulation of T-cells in some parts of the nucleus, particularly those closest to the graft.

### Insertion of a living peripheral nerve graft into the rubrospinal tracts induces regeneration of rubrospinal axons, activation of microglia around neurons with regenerating axons, but no accumulation of T-cells in the red nucleus

**Figure 9:**
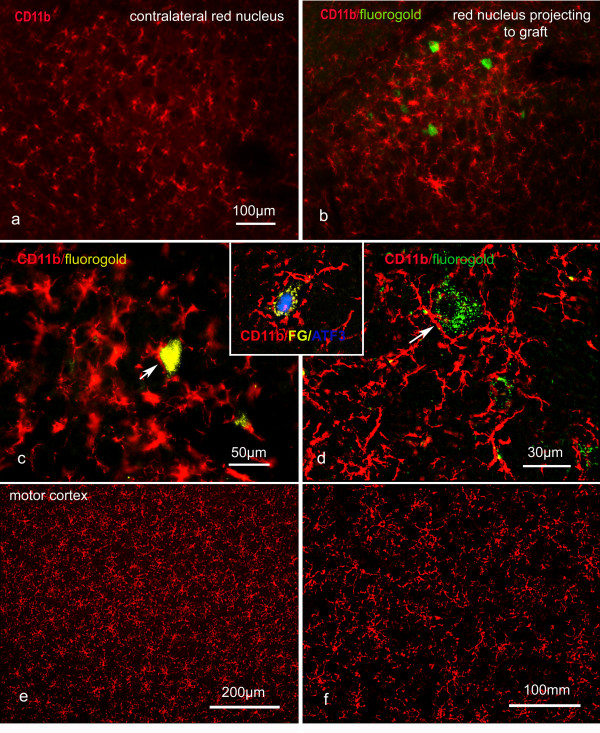
**Microglia in the red nucleus (a-d and inset) and motor cortex (e, f) 4 weeks after nerve graft implantation into the rubrospinal tract and cervical dorsal columns respectively**. Neurons with regenerating axons were retrogradely labelled with fluorogold. Microglia established close contacts with regenerating rubrospinal neurons (arrows), which also expressed ATF3 (inset). No retrogradely labelled corticospinal neurons were detected (e and f) and there was no microglial activation in the motor cortex. (a-c) are non-deconvolved and (d-f plus inset) are deconvolved images. The scale bar in (a) also applies to (b).

15 animals were killed 1 - 4 weeks after the implantation of a living nerve graft into the right rubrospinal tract. 3 animals were killed 3 weeks after grafting and subject to β-thymosin immunofluorescence, and 4 rats were killed at 1,2 and 4 weeks and subject to CD11b immunofluorescence. In 2 of 3 animals killed 3 weeks after insertion of a living graft and subject to β-thymosin immunofluorescence, and in all 4 rats killed 4 weeks after insertion of a living graft and subject to CD11b immunofluorescence, retrogradely labelled rubrospinal neurons were present, indicating that rubrospinal axons had regenerated through the grafts. All the rats with retrogradely labelled rubrospinal neurons showed a clear increase in the intensity of β-thymosin or CD11b staining of microglia and an apparent increase in the number of microglia in sections of the red nucleus containing regenerating neurons (Fig 9a-d). One rat with a 3 week graft but which did not have retrogradely labelled neurons, probably indicating that no axonal regeneration had taken place, did not show enhanced β-thymosin activity in either red nucleus.

The enhanced β-thymosin/CD11b activity was restricted to the red nucleus. In one of the animals that had regenerated axons the number of β-thymosin-positive microglia was counted: 294 microglia per mm^2 ^were present on the side containing the regenerating neurons compared to 227 microglia per mm^2 ^on the intact side. CD11b-positive microglia were counted in the red nucleus of rats at 1, 2 and 4 weeks (Fig. 4; n = 4 in each case). In every grafted animal the number of microglial cells was significantly greater in the red nucleus projecting to the graft in the 4 week animals (P = < 0.05, two-tailed paired t test). There was no significant difference between the operated and control sides for axotomy animals. No T-cells were found in the red nucleus of animals with nerve grafts in the rubrospinal tracts.

In 3 rats given freeze-killed grafts into the lateral column of the spinal cord and killed 4 weeks after operation, no retrogradely labelled neurons were found in the red nucleus and there was no increase in immunofluorescence for CD11b and no T-cell accumulation in the nucleus (data not shown).

Thus, there was a correlation between microglial activation (but not T-cell accumulation) in the red nucleus and regeneration of rubrospinal axons.

### Peripheral nerve graft insertion into the cervical corticospinal tracts does not induce axonal regeneration or inflammation in the motor cortex

In 5 rats 4 weeks after tibial nerve grafts were implanted into the dorsal columns of the spinal cord, and 3 days after fluorogold was applied to the distal end of the graft, no retrogradely labelled neurons were found in the motor cortex, confirming that corticospinal axons rarely regenerate into peripheral nerve grafts in the spinal cord. All these animals were subject to immunofluorescence for CD11b. No change in CD11b immunoreactivity was detected in the motor cortex of these animals (Fig. 9e and 9f) and 230.6 +/- 21 microglia mm^2 ^were identified in lamina V of the motor cortex of 5 unoperated rats and 245.3 +/- 22.3 microglia mm^2 ^were found in the motor cortex of rats with 4 week grafts. These values are not significantly different (P = > 0.3, 2-tailed t test).

There was no increase in the numbers of T-cells in the motor cortex in grafted animals. The numbers of T-cells in sections of the hind limb and fore limb cortex were counted in two of the rats with a nerve graft inserted into the dorsal corticospinal tract. 0.6 +/- 0.5 T-cells were identified per field of layer 5 in the motor cortex, a similar number to that found in unoperated animals (see above).

## Discussion

This study shows that there are marked changes in microglial morphology and the number of microglia around the cell bodies of intrinsic CNS neurons regenerating axons into a peripheral nerve graft. There were few morphological signs of perineuronal microglial activation when the corticospinal tracts or the rubrospinal tracts were cut and/or exposed to a peripheral nerve graft under conditions where regeneration did not occur. Hence the activation of microglia around the cell bodies of neurons was closely correlated with axonal regeneration of intrinsic CNS neurons and seems to require interactions between regeneration-competent axons and living peripheral nerve tissue. In contrast, perineuronal accumulation of T-cells was not reliably correlated with axonal regeneration by intrinsic CNS neurons.

### Perineuronal microglial activation is linked to axonal regeneration rather than axotomy alone

Major changes in microglial activation and a substantial increase in the number of perineuronal microglial, occur around motor neurons when they are regenerating axons after peripheral nerve injury [4,24,34] even in the absence of neuronal death. In contrast, the present study and many previous studies of axotomized intrinsic CNS neurons have reported little or no activation or increase in the number of perineuronal microglia after axotomy unless considerable neuronal cell death is induced [11,12,35,36]. There are no previous data on the microglial responses to TRN axotomy but varying degrees of microglial activation have been reported in the red nucleus after injuries to the rubrospinal tract in the spinal cord in adult rats [14,15]. This occurs predominantly on the axotomized side but to lesser extent in the "uninjured" nucleus (which contains a few ipsilaterally projecting neurons). However, a more pronounced microglial activation and proliferation can be induced by cell death of neurons in the red nucleus (following a very proximal rubrospinal axotomy) [37].

In the present study mechanical injury in the absence of a living nerve graft produced only variable and modest signs of microglial activation around axotomized intrinsic CNS neuronal cell bodies. Thus, even when regeneration-competent intrinsic CNS neurons (in the TRN or red nucleus) were subject to a distal axotomy, there was little perineuronal microglial activation in the absence of axonal regeneration, presumably indicating that there was also little neuronal cell death.

Inserting a peripheral nerve graft into the thalamus or cervical rubrospinal tract both axotomizes TRN or rubrospinal neurons and stimulates their regeneration. TRN neurons are probably the most successful intrinsic CNS neurons at regenerating axons into peripheral nerve grafts in the brain [27,28]; and the regeneration of their axons provoked the most obvious inflammatory response in the present study, including the partial enwrapping of the neuronal cell bodies. Axotomized rubrospinal neurons regenerated axons into a peripheral nerve graft in the cervical spinal cord in this and in previous studies [38,39] and in all the animals with retrogradely labelled neurons in the red nucleus there was a pronounced perineuronal microglial response. In contrast, corticospinal neurons did not regenerate axons into peripheral nerve grafts in the spinal cord in the present study, or in most previous studies [40,41], and there was no increase in microglial activity in the motor cortex in the animals receiving peripheral nerve grafts in the cervical dorsal columns. This shows that the exposure of injured intrinsic CNS axons to a living nerve graft is not in itself enough to induce a microglial response around the injured neurons; such a response must depend on interactions between regeneration-competent neurons and living peripheral nervous tissue.

### The lack of a microglial response to corticospinal axotomy

The absence of microglial activation in the motor cortex one week after unilateral pyramidectomy or 1-2 weeks following bilateral injury to the cervical dorsal corticospinal tracts is in keeping with the absence of a detectable cell body response by corticospinal neurons to spinal cord injury [42]. Both the present findings and those of Mason et al. [42] are perhaps surprising in view of the report that there is substantial apoptotic cell death of corticospinal neurons 7 days after following spinal cord injury [43]. Cell death of facial motor neurons after axotomy is accompanied by a clustering of activated microglia around the dying cells [44] and a similar phenomenon might have been expected following corticospinal cell death. However, other studies in rats [45-47], hamsters [48] and primates [49] have failed to detect substantial corticospinal cell death after axotomy in the spinal cord.

The forebrain is resistant to inflammation: application of bacterial lipopolysaccharide to the motor cortex in rats with a cervical spinal cord injury induced a transient inflammation around corticospinal neurons and a transient increase in some neuronal growth-related genes, but did not stimulate corticospinal sprouting or regeneration [50].

### Extent of microglial activation

The microglial interactions with regenerating TRN and rubrospinal neurons were not as intense or intimate as those seen around regenerating motor neurons. The processes of activated microglia enwrapped the cell bodies of regenerating facial nucleus neurons after axotomy of the facial nerve in this and previous studies [24,51,52]. The peak of this phenomenon occurs 4-7 days after nerve injury[24]. In contrast, in our study, relatively few regenerating TRN or rubrospinal neurons were completely encircled by microglial processes, although partial enwrapping was common. However, the survival time of the animals with retrogradely labelled (i.e. identified regenerating) TRN or red nucleus neurons was 3-4 weeks after grafting. It is possible that enwrapping of perikarya of regenerating neurons by microglia might have been a widespread phenomenon after shorter postoperative intervals and that the enwrapping had largely disappeared by the time the regenerating CNS neurons could be identified by retrograde labelling.

### The role of activated microglia in axonal regeneration

There are two likely explanations of the correlation between the extent of the microglial or macrophage response around axotomized neurons and their ability to regenerate axons. First it is possible that the microglia/macrophages stimulate the injured neurons to regenerate their axons [18,20]. Second, the microglia/macrophages may be involved in immune surveillance unrelated to regeneration [17,24]. The explanations are not, of course, exclusive and different forms of microglia activation may occur. The strong evidence that macrophages or microglia might stimulate axonal regeneration comes from experiments in which inflammation has been artificially induced around neuronal cell bodies. Injecting an inflammatory agent into dorsal root ganglia increases the rate of axonal regeneration in injured dorsal roots [18], presumably by stimulating the neuronal cell body response [53]. Similarly, injecting zymosan into the vitreous body increases inflammation in the retina, stimulates the cell body response to axotomy and enhances axonal regeneration in the optic nerve [20]. It has been suggested that macrophages in the eye secrete oncomodulin, which in turn stimulates retinal ganglion cells to regenerate their axons [54]. However, others have denied this [55] and there is some indication that stimulation of axonal regeneration is produced by a mixture of macrophage-derived and lens-derived factors [56].

It is clear that motor neurons capable of vigorous regeneration do not always require a pronounced macrophage or microglial response to do so. In mice with a frame-shift mutation in the macrophage-colony stimulating factor (M-CSF) gene [57], the microglial reaction to facial axotomy is muted and microglial processes do not enwrap facial neurons [24]. Studies using such mice or mice treated with the mitotic inhibitor, cytosine arabinoside [34], which prevents microglial proliferation, have found no change in neuronal survival or axonal regeneration. If the main role of microglia around axotomised neurons is immune surveillance [24], then the immune surveillance of intrinsic CNS neurons is enhanced during axonal regeneration compared with axotomy alone. It remains possible that microglial activation around the cell bodies of regenerating neurons plays some role in supporting the regenerative phenotype in neurons, and may be necessary for the regeneration of neurons with a relatively weak response to axotomy.

### Axonal regeneration by intrinsic CNS neurons is not associated with perineuronal accumulation of T-cells

T-cells accumulate near the cell bodies of motor, sensory and autonomic neurons after axotomy [7,8,16,17,58]. Each of these types of neuron is capable of vigorous axonal regeneration. T-cells did not, however, accumulate around axotomized rubrospinal or corticospinal cell bodies or around rubrospinal neurons when they were regenerating their axons. They were found near regenerating TRN neurons but were most frequent in those regions close to the nerve graft. It is not clear whether this represents a "spill-over" from the graft in which many T-cells were present or part of an inflammatory response to the regenerating neurons. T-cells are believed to have neuroprotective functions for axotomized motor neurons in mice [22,59,60]. It has been claimed that T-cells can have a neuroprotective role in the injured spinal cord [61]. However, T-cells are not always neuroprotective [62,63]. The present study showed an absence of T-cell accumulation around regenerating rubrospinal neurons. T-cells are not likely to be involved in stimulating axonal regeneration by CNS neurons when their axons are exposed to peripheral nerve grafts. Some authors have claimed that the T-cell response around axotomized motor neurons is absent or muted in rats [64,65]compared with mice, on which most studies have been performed. Our findings are in line with those of Olsson[66] in that a clear T-cell response was present in the rat facial nucleus following axotomy.

## Conclusions

Morphological signs of perineuronal microglial activation but not T-cell accumulation are closely associated with axonal regeneration by intrinsic CNS neurons. The molecular changes in such microglia are unknown but evidence from the visual system suggests that activated microglia/macrophages may be capable of stimulating axonal regeneration [20,56,67], possibly by the secretion of oncomodulin [54].

## Methods

### Surgery

Adult male or female Sprague-Dawley rats, weighing 200-250 g were used throughout. All procedures were approved by the UCL ethics committee and the UK Home Office. Rats were anaesthetized using a halothane-nitrous oxide-oxygen mixture. After the surgery the animals were injected subcutaneously with an antibiotic (Clamoxyl, SKB, 0.5 ml) and intramuscularly with an analgesic (Buprenorphine 0.05 mg/kg).

### Peripheral nerve graft or stab wound in thalamus

A 2 cm long piece of tibial nerve was removed from the left hind limb, and inserted into the thalamus through a craniotomy, 4.5 mm caudal to the bregma and 2.5 mm lateral from the midline. The tibial nerve graft was pushed 7 mm deep using a micropipette, and then secured to the skull using Histoacryl (B. Brawn, Aesculp, Germany) glue. The distal end of the graft was left blind ended on top of the skull. Both the wound on the hind limb and the skull were closed with sutures. Freeze-killed nerve grafts were produced by placing the nerve segment on aluminium foil and putting it through 6 cycles of freezing and thawing using dry ice. To produce a stab wound a needle of similar thickness to the tibial nerve was inserted to the same co-ordinates as the nerve grafts.

### Corticospinal tract transection, rubrospinal tract transection, pyramidotomy and peripheral nerve graft insertion in the spinal cord

A laminectomy was carried out at the level of C3 to expose the spinal cord. The vertebral dura was opened. To section the rubrospinal tract the lateral white column was cut on the right side with microsurgical scissors. To section the dorsal corticospinal tract the dorsal columns were transected bilaterally, the lesion passing as deep as the central canal. For animals which had a peripheral nerve graft inserted, a 2 cm segment of the peroneal nerve taken from the left hind limb was inserted into the right lateral white column or into the midline of the dorsal columns at C3 with the distal end of the graft secured subcutaneously with an 8/O suture. In three animals the peroneal nerve was put through 4 cycles of freeze-thawing on dry ice before grafting into the lateral column of the spinal cord. The left peroneal nerve was cut in all spinal transection experiments, to control for the injury inflicted during nerve grafting. Pyramidotomy was performed by exposing the medullary pyramids through a bur hole in the ventral part of the occipital bone and severing the right pyramid with microsurgical scissors.

### Retrograde labelling

In some experiments cholera-toxin B (CTB; List Biological Laboratories; 1 μl of 1% solution in sterile water) was injected into the distal end of the graft 2-3 days before the rats were sacrificed. In other experiments 1 μl 4% Fluorogold was applied to the distal end of the graft. Fluorogold was applied to the site of injury as retrograde tracer in rubrospinal and corticospinal lesion-only experiments. In some experiments ATF3 immunostaining was used to identify axotomized rubrospinal neurons.

The rats were killed by overdose with Halothane or pentobarbitone and then perfused with 200 ml 0.1 M phosphate buffered saline (PBS) followed by 500 ml 2% paraformaldehyde in phosphate buffer pH 7.4.

### Immunostaining of sections

Sections were cut at 40 μm on a freezing-microtome and reacted for antigens as described previously [50]. The antibodies used in different experiments were as follows. β-thymosin for microglia (rabbit anti Xenopus thymosin β4 [68] 1:500); OX-42 for microglia (monoclonal, Serotec, Oxford, UK 1: 1000; CTB (goat polyclonal 1:100,000 - List Biological Laboratories, CA, USA. 1:60,000); CD6 for T-cells (OX52; mouse monoclonal, Serotec, Oxford UK 1:300); Anti TCR for T-cells (mouse monoclonal, Serotec, Oxford UK 1:1000); ATF3 (rabbit polyclonal, Santa Cruz, CA, USA 1:800). All were applied overnight at 4°C. The appropriate biotinylated secondary was used at a concentration of 1:300. Tyramide signal amplification (Kit supplied by NEN) was performed in accordance with the manufacturer's instructions to visualize CTB.

### Analysis of results

Micrographs of β-Thymosin stained sections were taken using a Leica confocal LEITZ DM R microscope or a Zeiss confocal LSM 510 META microscope. Other micrographs were taken using a Hamamatsu Orca digital camera attached to a Zeiss Axioplan microscope. Some sections were deconvolved using Openlab software. Dark field observations allowed the boundaries of the nuclei to be ascertained.

Cell counting. The number of β-Thymosin stained microglial cell bodies in the TRN was counted in 4 micrographs containing retrogradely labelled neurons, and equivalent areas of the contralateral side, taken using the 40× objective. For rubrospinal transection or graft experiments, 4 sections identified as containing rubrospinal neurons by retrograde labelling or ATF3 immunoreaction were used to obtain micrographs showing microglia in the red nucleus. Images from control unoperated animals (therefore lacking retrograde labelling or ATF3) were matched to the experimental sections morphologically. The area of the red nucleus used for counting in each section was measured using Openlab software. The images of the experimental and contralateral sides were always taken at the same time and with the same settings.

T-cells were difficult to resolve in low power micrographs and were therefore identified using the ×40 objective and mapped onto low power dark field images of the appropriate part of the brain. The cells were counted in four 40 μm sections per animal. The boundaries of the facial nucleus are easily identified in dark field images and the area measured using Openlab software. The motor cortex was identified by retrograde labelling of corticospinal neurons (axotomy without a graft experiments), or in experiments where there was no retrograde labelling, in morphologically equivalent areas (ATF3 was not expressed by axotomized corticospinal neurons). Layer V was identified on morphological criteria in DAPI stained sections or in equivalent regions in fluorogold-labelled sections.

## Authors' contributions

Nerve grafting experiments were performed by GC and PNA and pyramidotomies by KT. Immunohistochemistry for CTB and β Thymosin in the TRN and red nucleus was performed by BNS, KT and BZYW. Most OX42 immunohistochemistry was performed by PNA. Most T-cell immunohistochemistry was performed by SS. All authors helped with the analysis of results. BNS produced the first draft of the manuscript, which was then critically revised and modified by PNA and ARL. All authors read and approved the final manuscript.
